# Distinct Microglial Responses in Two Transgenic Murine Models of TAU Pathology

**DOI:** 10.3389/fncel.2018.00421

**Published:** 2018-11-14

**Authors:** Carmen Romero-Molina, Victoria Navarro, Raquel Sanchez-Varo, Sebastian Jimenez, Juan J. Fernandez-Valenzuela, Maria V. Sanchez-Mico, Clara Muñoz-Castro, Antonia Gutierrez, Javier Vitorica, Marisa Vizuete

**Affiliations:** ^1^Departamento Bioquimica y Biologia Molecular, Facultad de Farmacia, Universidad de Sevilla, Seville, Spain; ^2^Instituto de Biomedicina de Sevilla, Hospital Universitario Virgen del Rocio, CSIC, Universidad de Sevilla, Seville, Spain; ^3^Centro de Investigacion Biomedica en Red sobre Enfermedades Neurodegenerativas, Madrid, Spain; ^4^Departamento Biologia Celular, Genetica y Fisiologia, Facultad de Ciencias, Instituto de Investigación Biomédica de Málaga (IBIMA), Universidad de Málaga, Málaga, Spain

**Keywords:** Alzheimer disease, microglia, tau models, inflammation, tauopathies

## Abstract

Microglial cells are crucial players in the pathological process of neurodegenerative diseases, such as Alzheimer’s disease (AD). Microglial response in AD has been principally studied in relation to amyloid-beta pathology but, comparatively, little is known about inflammatory processes associated to tau pathology. In the hippocampus of AD patients, where tau pathology is more prominent than amyloid-beta pathology, a microglial degenerative process has been reported. In this work, we have directly compared the microglial response in two different transgenic tau mouse models: ThyTau22 and P301S. Surprisingly, these two models showed important differences in the microglial profile and tau pathology. Where ThyTau22 hippocampus manifested mild microglial activation, P301S mice exhibited a strong microglial response in parallel with high phospho-tau accumulation. This differential phospho-tau expression could account for the different microglial response in these two tau strains. However, soluble (S1) fractions from ThyTau22 hippocampus presented relatively high content of soluble phospho-tau (AT8-positive) and were highly toxic for microglial cells *in vitro*, whereas the correspondent S1 fractions from P301S mice displayed low soluble phospho-tau levels and were not toxic for microglial cells. Therefore, not only the expression levels but the aggregation of phospho-tau should differ between both models. In fact, most of tau forms in the P301S mice were aggregated and, in consequence, forming insoluble tau species. We conclude that different factors as tau mutations, accumulation, phosphorylation, and/or aggregation could account for the distinct microglial responses observed in these two tau models. For this reason, deciphering the molecular nature of toxic tau species for microglial cells might be a promising therapeutic approach in order to restore the deficient immunological protection observed in AD hippocampus.

## Introduction

Alzheimer’s disease (AD), the most common cause of dementia in the elderly, is pathologically characterized by the presence of extracellular amyloid-beta (Abeta) plaques and intraneuronal neurofibrillary tangles (NFTs) of highly phosphorylated tau protein. Besides these two types of protein lesions, AD pathology includes loss of synapses and selective neuronal cell death, along with a chronic inflammatory response manifested by activated microglia and astrocytes (for recent review Heppner et al., 2015; Calsolaro and Edison, 2016; Ransohoff, 2016; Cuello, 2017). Microglial cells control the immune response in the brain. Any insult that disrupts brain homeostasis is rapidly detected by microglia, that become activated manifesting some typical features as migration to the injury site, morphological alterations, and changes in the gene expression profile (Hanisch and Kettenmann, 2007; Wolf et al., 2017; Li and Barres, 2018). The role of microglia in AD has been mainly studied in relation to the amyloid pathology in both patients and animal models (Jimenez et al., 2008; Prokop et al., 2013; Serrano-Pozo et al., 2013; Guillot-Sestier et al., 2015; Sarlus and Heneka, 2017; Hansen et al., 2018). However, microglial cells have also been implicated in tau pathology during AD pathogenesis (for review, Laurent et al., 2018).

The involvement of microglia as crucial players in the progression of AD has recently been estimated (Calsolaro and Edison, 2016; Ransohoff, 2016; Sarlus and Heneka, 2017). Microglia seem to have an active and biphasic role on the AD pathology, promoting at early stages the clearance of Abeta deposits (Condello et al., 2015) and, at late stages, mediating the loss of synapses (Wu et al., 2015; Spangenberg and Green, 2017), the exacerbation of the Abeta and tau pathologies (Xiang et al., 2016; Leyns and Holtzman, 2017) and the secretion of reactive oxygen species and inflammatory cytokines (Colonna and Butovsky, 2017). Therefore, it remains doubtful if activated microglia have a protective and/or detrimental effect in the progression of AD pathology. Thus, a therapeutic possibility for AD, targeting microglia, requires a fully understanding and a better characterization.

Broad single-cell RNA sequencing (RNA-seq) analysis in the 5XFAD model of AD identified a new and singular disease-associated microglia (DAM) associated to amyloid plaques (Keren-Shaul et al., 2017). These ‘DAM’ microglia are molecularly characterized by expressing typical microglial markers, such as *Iba1*, *Cst3*, and *Hexb*, coupled with downregulation of “homeostatic” microglial genes (*P2ry12*, *Cx3cr1*, *CD33*, and *Tmem119)* (Butovsky et al., 2014). DAM cells further display upregulation of several genes identified as AD risk factors, such as *Apoe*, *Ctsd*, *Lpl*, *Tyrobp*, and *Trem2* (Lambert et al., 2013). Interestingly, DAM phenotype has also been identified in other amyloidogenic AD mouse models (Krasemann et al., 2017; Ofengeim et al., 2017; Mrdjen et al., 2018), in tauopathy models (Leyns and Holtzman, 2017; Friedman et al., 2018) and in other neurodegenerative diseases, such as amyotrophic lateral sclerosis (Spiller et al., 2018) or multiple sclerosis (Krasemann et al., 2017), as well as in aging (Mrdjen et al., 2018; Olah et al., 2018). Nevertheless, the mechanisms that regulate the microglia phenotype in disease are not known.

Aging or genetic susceptibility may lead to impaired microglia function (Streit et al., 2009, 2014). TREM2 has been shown to have proliferative and pro-survival functions in microglia (Otero et al., 2009; Ulland et al., 2017). It has recently been demonstrated that the TREM2-APOE pathway induces a microglia phenotypic switch from a homeostatic to neurodegenerative profile (Krasemann et al., 2017). In this sense, several mutations in TREM2 (such as R47H TREM2) produce an increased risk for late-onset AD (Yeh et al., 2016) and impair the microglial signals involved in survival, proliferation, chemotaxis, and phagocytosis (Jay et al., 2017; Ulrich et al., 2017; Yeh et al., 2017). Moreover, deficiencies in TREM2 are also associated with rare hereditary neurodegenerative diseases (Paloneva et al., 2002; Chitu et al., 2016). In both diseases, microglial response and, more relevant, microglial survival seems to be compromised (Konno et al., 2014; Tada et al., 2016).

Opposite to the strong microglial activation in amyloidogenic mice, we have recently demonstrated a significant microglial degenerative process in the hippocampus of AD patients (Sanchez-Mejias et al., 2016; Gutierrez and Vitorica, 2018; Navarro et al., 2018), a brain region with a more prominent tau pathology than Abeta accumulation. We have also confirmed that soluble phospho-tau was, at least in part, responsible for this degenerative process. In the present study, we analyzed the hippocampal microglial response in the context of tau pathology. For this purpose, we compared two different transgenic mouse models, ThyTau22 and P301S. While the hippocampus of ThyTau22 animals manifested attenuated microglial activation similar to AD patients, there was a clear microglial response in the P301S model, reflecting remarkable differences in the innate immune response to tau pathology.

## Materials and Methods

### Mouse Models

Animal experiments were performed in accordance with the Spanish and the European Union regulations (RD53/2013 and 2010/63/UE) and approved by the Animal Research Committees from the Universities of Seville and Malaga (Spain). For this study, 9–18 month-old APP751sl, 2–4 and 9–12 month-old ThyTau22 (commercialized by Sanofi), and 2–4 and 9–12 month-old P301S (Jackson Laboratory) transgenic animals, and age-matched wild-type mice (C57BL/6) were used (males and female where used indifferently). The APP mice over-expressed the human APP751 carrying the Swedish (KM670/671NL) and London (V717I) mutations. The ThyTau22 mice expressed human 4-repeat tau with G272V and P301S mutations under a Thy1.2 promotor. The P301S mice expressed human 4-repeat 1 N-terminal insert tau with P301S mutation driven by the mouse prion protein (Prnp) promoter (B6;C3-Tg(Prnp-MAPT^∗^P301S)PS19Vle/J). Mice were anesthetized (sodium pentobarbital, 60 mg/kg) and processed as described (Jimenez et al., 2008; Sanchez-Varo et al., 2012; Torres et al., 2012).

### Antibodies

The following primary antibodies were used: anti-phospho-tau AT100 (pSer212/Thr214, #MN1060, Thermo Fisher Scientific), AT8 (MpSer202/Thr205, #MN1020, Thermo Fisher Scientific), AT180 (Thr231, #MN1040, Thermo Fisher Scientific); anti-total tau (Tau46, #4019S, Cell Signaling Technology and Tau12 #MAB2241, Millipore); anti-b-actin (#A5316, Sigma-Aldrich), anti-CD45 (IBL-3/16, Bio-Rad), Anti-Iba1 (#019-19741, Wako Pure Chemical Industries), Anti-CD68 (#125212, Abcam), anti-caspase 3 (#9661 Cell Signaling Technology), anti-caspase 8 (#9429, Cell Signaling), anti-caspase 9 (#9507, Cell Signaling). As secondary antibodies, we used HRP anti-mouse (#7076S, Cell Signaling) and HRP anti-rabbit (#7074S, Cell Signaling).

### Preparation of S1 Soluble Fractions

Soluble fractions (S1) from mice brain cortex were prepared as described (Jimenez et al., 2014). Briefly, mouse tissue was homogenized using Dounce’s homogenizer, in PBS containing protease and phosphatase inhibitors (Roche). Homogenates were ultracentrifuged at 4°C for 60 min, at 100,000 × *g* (OptimaMAX Preparative Ultracentrifuge, Beckman Coulter). Supernatants (S1 fractions), which comprise both extracellular and cytosolic proteins, were aliquoted and stored at -80°C until further use. The pellets (P1) were processed using two different protocols: A) P1 were sonicated applying 8 pulses of 15 s each and then centrifuged 27,000 × *g* 4°C, 15 min. The supernatant obtained (S1s) was aliquoted and stored at -80°C until further use; B) P1 were extracted in RIPA buffer (1% CHAPS, 1% Na-deoxycholate, 0.2% SDS, 140 mM NaCl, 10 mM Tris–HCl, pH 7.4); ultracentrifuged and supernatants, S2 fractions (intracellular particulate proteins), were stored. Pellets (P2) were re-extracted in buffered-SDS (2% SDS in 20 mM Tris–HCl, pH 7.4, 140 mM NaCl), centrifuged as above and supernatants, S3 (SDS releasable proteins) were stored at -80°C.

### Sarkosyl-Insoluble Fraction Isolation

Sarkosyl-insoluble tau was isolated as described (Luo et al., 2015). Mouse hippocampi were homogenized in “homogenization buffer” (10 mM Tris, 0.8 M NaCl, 1 mM EGTA, 10% sucrose, pH 7.4, plus protease and phosphatase inhibitors). Homogenates were ultracentrifuged at 4°C for 60 min, 5,000 × *g*. Then, supernatants were incubated for 2.5 h at 37°C in agitation with 1% Sarkosyl (Sigma-Aldrich) and 1% beta-mercaptoethanol (Sigma-Aldrich) in “homogenization buffer” followed by ultracentrifugation (4°C, 30 min, 100,000 × *g*). Supernatants constituted the Sarkosyl soluble fractions and pellets were the Sarkosyl-insoluble fraction. This insoluble fraction was rinsed twice and resuspended in TBS.

### Total RNA and Protein Extraction

RNA and proteins were isolated from mouse hippocampal tissue using TriPure Isolation Reagent (Roche). RNA was first purified and quantified using NanoDrop 2000 spectrophotometer (Thermo Fischer). Proteins were then isolated, resuspended in 4% SDS, 8M Urea, and quantified using Lowry’s method.

### Retrotranscription and Quantitative Real-Time RT-PCR

Retrotranscription (RT) of 4 μg of total RNA was performed with the High-Capacity cDNA Reverse transcription Kit (Applied Biosystems). For real-time qPCR, 40 ng of cDNA were mixed with Eagle Taq Master Mix (Sigma) and Taqman Gene Expression assay probes (Applied Biosystems). The following genes were analyzed, CD45 (ref. Mm01293577_m1), CD68 (ref. Mm03047340_m1), Clec7a (Mm01183349_m1), CX3CR1 (Mm02620111_s1), GAPDH (ref. Mm99999915_g1), Iba1 (ref. Mm00479862_g1), IGF-1(ref Mm00439560_m1), P2ry12 (ref. Mm01950543_s1), and TMEM119 (ref. Mm00525305_m1). Quantitative PCR reactions (qPCR) were done using an ABI Prism 7900HT (Applied Biosystems). Results were expressed using the comparative double-delta Ct method (2-ΔΔCt). ΔCt values represent GAPDH normalized expression levels.

### Western Blots

Western blots were performed as previously described (Jimenez et al., 2008). To analyze the different forms of Tau, protein samples were loaded onto 4–20% SDS-Tris–Glycine-PAGE (Bio-Rad). For the rest of the studied proteins, we used 12% SDS-Tris–Glycine-PAGE. In all cases, proteins were transferred to nitrocellulose membranes (Optitran, GE Healthcare Life Sciences). Membranes were blocked using 2% low-fat milk in TPBS (0.1% Tween-20, 137 mM NaCl, 2.7 mM KCl, 10 mM phosphatebuffer, pH 7.4) and incubated overnight, at 4°C, with the appropriate primary antibody. Anti-mouse or anti-rabbit horseradish-peroxidase-conjugated secondary antibodies were used in each case at a dilution of 1/10.000. The blots were developed using the Pierce ECL 2 Western Blotting Substrate detection method (0.5 pg, lower limit sensitivity; ThermoScientific). The images were obtained and further analyzed with ChemiDoc^TM^ Touch Imaging System (Bio-Rad).

### Immunohistochemistry

Fixed (4% paraformaldehyde) free-floating coronal brain sections (40 μm thickness) from 9 month-old ThyTau22 and P301S mice were assayed. After antigen retrieval (80°C for 20 min in 50 mM citrate buffer, pH 6.0), endogenous peroxidase was inhibited (3% H2O2/10% methanol in PBS, pH 7.4 for 20 min) and non-specific staining was avoided using 5% goat serum (Sigma-Aldrich) in PBS. Sections were incubated with the primary antibody (overnight at room temperature) followed by the corresponding biotinylated secondary antibody (1:500 dilution, 1 h at room temperature, Vector Laboratories), streptavidin-conjugated horseradish peroxidase (1:2000, 90 min, Sigma-Aldrich), and visualized with 0.05% 3-3-diaminobenzidine tetrahydrochloride (DAB, Sigma-Aldrich) and 0.01% hydrogen peroxide in PBS. The specificity of the immune reactions was controlled by omitting the primary antisera. Sections were examined under a Nikon Eclipse 80i microscope and images were acquired with a Nikon DS-5M digital camera using the ACT-2U imaging software (Nikon Corporation).

### Cell Cultures

BV2 microglial cells were grown (37°C and 5% CO2) in RPMI 1640, 2 mM glutamine, 10% (v/v) fetal bovine serum, plus penicillin/streptomycin (all from Biowest). For experiments of BV2 treatment with S1 soluble fractions, BV2 cells (15.000 cells/cm2) were serum deprived and treated with staurosporine 1uM (#S6942, Sigma) or S1 fractions (0.1 μg/μl) for 3, 6, and 12 h. When inhibiting caspases, 40 μM z-VAD-FMK (#V116, Sigma) were added 30 min before S1 treatment.

### Flow Cytometry Analysis of Cell Viability

The viability of BV2 cells was assessed using the apoptosis detection kit Annexin V-FITC (Immunostep) following the manufacturer’s specifications and was analyzed using a FACS Canto II flow cytometer (BD Services, San Jose, CA, United States).

### Statistical Analysis

Data were expressed as the mean ± SD (GraphPad). Mean values were compared using ANOVA followed by Tukey’s test (more than two groups) or two-tailed *t*-test (for two group comparisons). The significance was set at 95% of confidence.

## Results

### Distinct Microglial Responses in the Hippocampus of Mutant Transgenic Tau Mice

We have previously reported the existence of a different microglial response between Abeta and Tau-based mouse models (Sanchez-Mejias et al., 2016). In fact, Abeta models, such as APP or APP/PS1 transgenic mice, presented clear and strong microglial activation, as determined by gene expression or immunohistochemistry, whereas the tested Tau model (ThyTau22) displayed low microglial activation (Sanchez-Mejias et al., 2016; Navarro et al., 2018). In order to ascertain the limited microglial response in Tau-based models, we have directly compared the expression of different microglial genes in the hippocampus of two Tau transgenic mice: ThyTau22 and P301S mouse models. Furthermore, we have also included an APP-model as positive control.

**FIGURE 1:**
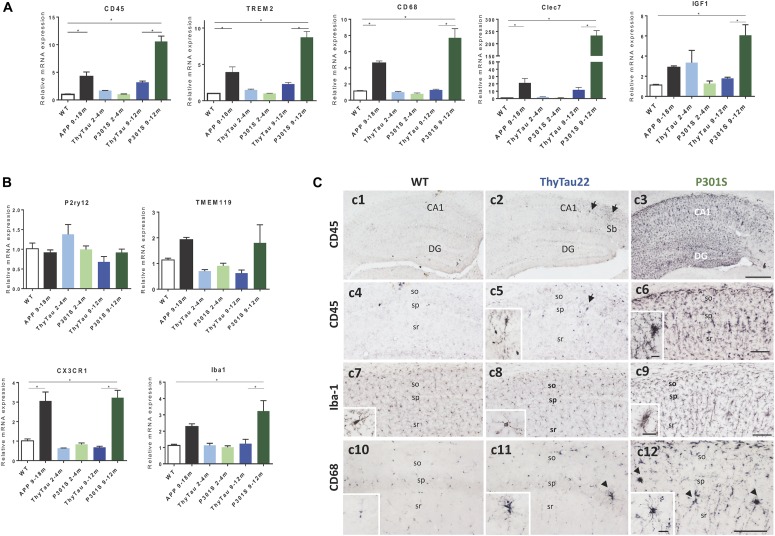
**(A,B)** Microglial DAM **(A)** and homeostatic **(B)** gene expression (qPCR) in ThyTau22 (*n* = 4–8/age) and P301S (*n* = 5–9/age) mice. Expression levels, normalized using GAPDH, were referred to WT mice [9 months-old WT (*n* = 4)]. APP mice (9–18 months, *n* = 4–7) were also included as positive control. ^∗^*p* < 0.05. Significance was analyzed by ANOVA followed by Tukey multiple comparison test. **(C)** Representative CD45 **(c1–c6)**, Iba-1 **(c7–c9)** and CD68 **(c10–c12)** -immunostained hippocampal sections from 9 month-old WT **(c1, c4, c7,** **c10)**, ThyTau22 **(c2, c5, c8,** **c11)** and P301S **(c3, c6, c9,** **c12)** mice. DG, debate gyrus; so, stratum oriens; sp, stratum pyramidale; sr, stratum radiatum. Scale bars, **(c1–c3)** 400 μm; **(c4–c12)** 100 μm; insets 20 μm.

First, we evaluated, by qPCR, the expression of microglial genes identified as DAM (disease associated microglia) program. As expected (Figure 1A), the expression of microglial activation markers (such as CD45, CD68, TREM2, Clec7a, and IGF-1) was significantly increased in the APP model (as compared to WT). Also, as previously reported (Sanchez-Mejias et al., 2016), ThyTau22 model displayed a limited microglial response at both, early (2–4 months) and advanced (9–12 months) ages. However, unexpectedly, we did observe strong microglial activation in the P301S model. In fact, the expression of all tested genes was even higher than that observed in our APP-based model. This strong response was restricted to 9–12 month-old P301S mice.

On the other hand, it has been reported that the microglial activation was paralleled by a decrease in the expression of the homeostatic genes (Keren-Shaul et al., 2017). Thus, we have also determined the expression of some homeostatic genes in the same animal cohorts. As shown, no differences were found in P2ry12 or TMEM119 whereas a significant increase was observed in CX3CR1 and Iba1 (Figure 1B). This increase may be due to an increment in the number of microglia cells, suggesting a possible proliferation of microglial cells upon activation (Askew et al., 2017; Baglietto-Vargas et al., 2017; Tay et al., 2017).

The microglial response in these two different Tau-models was also assessed by immunohistochemistry using CD45, Iba1, and CD68 antibodies. As shown in Figure 1C, most of microglial cells in the hippocampus of the P301S model were clearly positive for the marker of activated microglia CD45 (Figures 1C,c3,c6). In fact, the microglial cells from all hippocampal fields were strongly immunopositive showing an activated morphotype characterized by hypertrophic cell body and short processes. However, in parallel experiments, the ThyTau22 mice barely showed any CD45-positive cells (Figures 1C,c2,c5). As expected, Iba1-positive microglia was normally present in both models (Figures 1C,c8,c9) and the morphological changes, related to activation, were easily detected in the P301S but hardly in ThyTau22 microglial cells, which mostly displayed a resting phenotype similar to WT mice (Figures 1C,c7). Finally, an additional marker specific for activated microglia with phagocytic activity, CD68 (Figures 1C, c10–12), exhibited a immunostaining pattern similar to CD45, confirming the above findings.

Therefore, these results demonstrated the existence of a strong microgliosis in Tau-based models. Nonetheless, this microglial response was clearly different depending on each Tau transgenic line.

### P301S Mice Showed Stronger Tau Pathology Than ThyTau22 Mice

**FIGURE 2:**
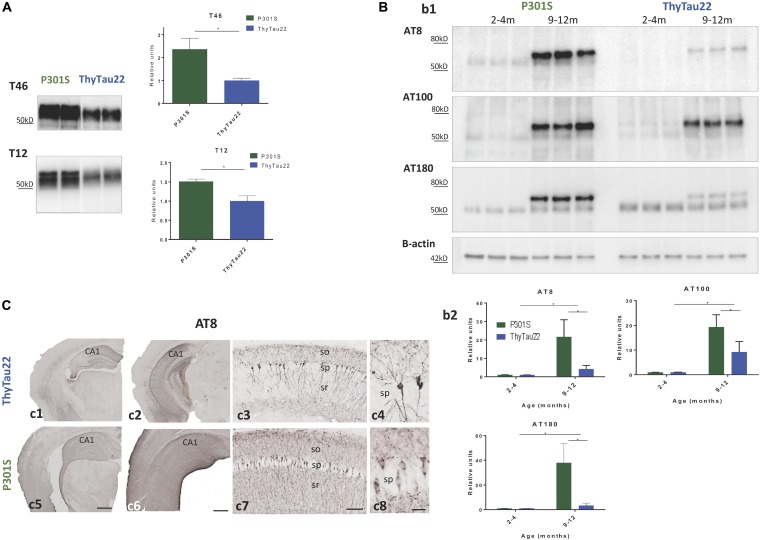
**(A)** Total tau (T46 upper panel and T12 lower panel) protein levels in 9 month-old P301S and ThyTau22 mice (*n* = 4–6). **(B)** Phospho-tau (AT8, AT100, AT180 antibodies) protein levels in P301S and ThyTau22 mice (*n* = 6). **(b1)** Representative western-blots. **(b2)** WB quantification, ^∗^*p* < 0.05, significance was analyzed by *t*-test between young and aged mice in each model and between aged P301S and ThyTau22 mice. **(C)** Representative AT8-immunostained hippocampal sections from 9 month-old ThyTau22 **(c1–c4)** and P301S **(c5–c8)** mice. so, stratum oriens; sp, stratum pyramidale; sr, stratum radiatum. Scale bars, **(c1**, **c2**, **c5**, **c6)** 500 μm, **(c3**, **c7)** 100 μm, **(c4**, **c8)** 20 μm.

An increase in the microglial response could be due to a different Tau expression and/or phosphorylation between these two models. Thus, we next directly tested the accumulation of total Tau in both models by western-blot using Tau46 (C-terminal) and Tau12 (N-terminal) antibodies. As expected, significant higher levels of total Tau were observed in P301S mice when compared to ThyTau22 mice (see Figure 2A).

This different tau accumulation could also produce a different phosphorylation pattern between both models. Therefore, we indirectly tested this possible difference using three phospho-specific monoclonal antibodies (AT8, AT100, and AT180), by western-blot. As expected (see Figure 2B), we observed an age-dependent increase in tau phosphorylation in both models. As shown, young (2–4 months of age) ThyTau22 or P301S mice displayed low (below the detection limits) phospho-tau accumulation. However, at 9–12 months of age, both ThyTau22 and P301S mice showed a significant accumulation in phospho-tau species, positive for all tested antibodies. In consonance with the high Tau expression, P301S model presented higher phospho-tau species, when compared with ThyTau22 mice (Figure 2B).

In addition, we verified the differences in tau phosphorylation by immunohistochemistry. We compared AT8-inmunostained sections from 9 month-old ThyTau22 and P301S mice. As shown in Figure 2C, in both Tau models the accumulation of phospho-tau, visualized by AT8 immunostaining, in the hippocampal region was notably higher when compared to other adjacent brain regions as cerebral cortex or thalamus (Figures 2C,c1,c5 rostral levels; Figures 2C,c2,c6 caudal levels). AT8 immunolabeling in the ThyTau22 hippocampus was associated with principal cells depicting neuronal perikarya and proximal dendrites in the CA1 region and dentate gyrus (Figures 2C,c3 and detail in Figures 2C,c4). Noteworthy, P301S hippocampus showed stronger neuropile phospho-tau immunostaining than ThyTau22 hippocampus. The stratum oriens and stratum radiatum of CA1 region displayed a widespread labeling, and numerous principal cell perikarya from stratum pyramidale appeared immunopositive (Figures 2C,c7 and detail in Figures 2C,c8).

Thus, these results suggest that the P301S model exhibits a higher tau pathology compared to the ThyTau22 model, which could explain the higher microgliosis reported.

### Soluble Phospho-Tau From P301S and ThyTau22 Mice Exerts Different Toxic Effect Upon Microglial cells

We have recently reported the toxic effect of soluble phospho-tau fraction on microglial cells *in vitro* (Sanchez-Mejias et al., 2016). Therefore, a higher expression and accumulation of phospho-tau species should produce higher microglial toxicity. Thus, we directly compared the toxic effect, on BV2 microglial cells, of soluble phospho-tau isolated from either 12 month-old ThyTau22 or P301S cerebral cortex. These soluble fractions (S1) comprised both extracellular and cytosolic proteins.

**FIGURE 3:**
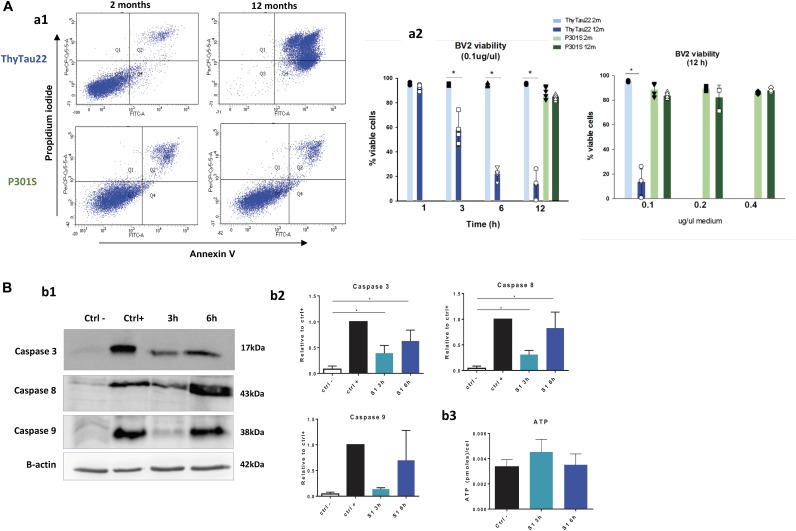
**(A)** Soluble fractions (S1) from aged ThyTau22 mice were toxic for BV2 microglial cells whereas S1 from aged P301S mice had no effect in the viability of microglial cells. **(a1)** Representative Annexin V/propidium iodide (PI) double staining of BV2 cells treated with S1 fractions at 0.1 ug/ul for 12 h. **(a2)** Quantitative analysis of cell viability after exposure to S1 fractions. Data is shown as the mean + SD (*n* = 3–5). ^∗^*p* < 0.05. Significance was determined by ANOVA and Tukey post-hoc test. **(B)** Caspase activation following microglial treatment with soluble phospho-tau from 12 month-old ThyTau22 mice. **(b1)** Representative western blots showing caspase 3, 8, and 9 activation in BV2 cells treated with S1 from aged ThyTau22 mice (0.1 ug/ul). B-actin (low panel) was used as the loading control.**(b2)** The relative abundance of cleaved caspases was determined by densitometry analysis. Data were normalized by positive control (staurosporine 1 μM), and significance was determined by ANOVA and Tukey post-hoc test (*n* = 3).**(b3)** No differences in ATP levels were observed in BV2 cells treated with S1 fractions from 12 month-old ThyTau22 mice (0.1 ug/ul). Data is shown as the mean + SD (*n* = 3).

First, we tested the effect of S1 fractions from ThyTau22 mice after 3, 6, and 12 h of treatment. As shown (Figure 3A) the S1 from 12 month-old ThyTau22 mice produced a clear and significant reduction in the number of viable BV2 cells, as compared to 2 month-old ThyTau22 mice (see also Sanchez-Mejias et al., 2016). After 12 h of treatment with S1 fractions, a significant low percentage of cells (13.7 ± 10.4%) remained viable. This pro-apoptotic effect was also ascertained by testing caspase activation. It is widely accepted that an increment in the cleavage of caspase 3 (17 kDa fragment) could reflect the apoptosis activation process. On the other hand, the cleavage of caspase 8 (43 kDa) or caspase 9 (38 kDa) was specific for the extrinsic or the intrinsic pathway, respectively. As shown in Figures 3B,b1,b2, S1 fractions derived from ThyTau22 models produced a clear and significant activation of caspases 8 and 3 and a non-significant increase of caspase 9, after 3 and 6 h of treatment. These results suggested that microglial cell death after treatment with soluble phospho-tau, was probably due to the activation of apoptotic pathways.

To support this finding, we also assessed the protective effect of a pan-caspase inhibitor (z-VAD-FMK) over the toxicity of 12-month old Thytau22 S1 fractions. As expected, in presence of z-VAD-FMK, the microglial viability was preserved (from 16 ± 8.8% to 83 ± 17.1% of viable cells after 6 h of treatment; *n* = 3).

Additionally, in order to evaluate if this microglial death was due to a depletion of ATP reservoirs, due to strong cell activation, ATP levels were measured in BV2 cells treated with S1 fractions from 12 month-old ThyTau22 mice. No changes were observed in ATP levels (see Figures 3B,b3), which may suggest that cell apoptosis is not trigger by a depletion of ATP pool.

On the other hand, we also tested whether similar soluble (S1) fractions isolated from P301S were also toxic for BV2 cells. Surprisingly (see Figures 3A,a1,a2), no toxicity was observed even after 12 h of treatment. In order to evaluate if this difference was due to a lower toxic dose of the soluble fractions, BV2 cells were treated with higher concentrations of S1 fractions (0.2 and 0.4 ug/ul) from 12 month-old P301S mice. As shown in Figures 3A,a2, even after a four-fold increase in the S1 dosage, no decrease in microglial viability could be observed.

### Different Tau Aggregation in Tau-Based Mouse Models

So far, our results demonstrated the different microglial response and toxicity between two Tau-based tg models. The strong microglial response observed in P301S, as compared with ThyTau22, could be a direct consequence of the higher Tau accumulation, both total and phosphorylated, observed in this particular model. However, this fact could not explain the non-toxic versus toxic effect of soluble phospho-tau on the microglial viability. Therefore, it is also possible the existence of a different aggregation pattern between both models.

**FIGURE 4:**
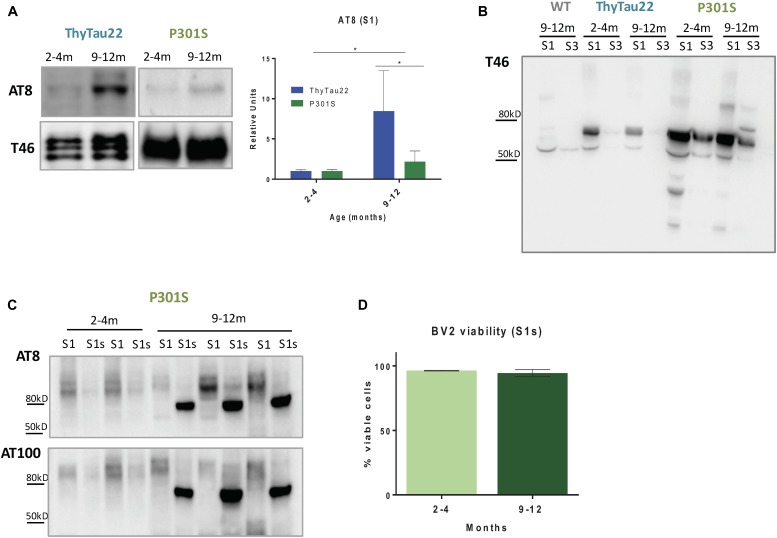
**(A)** Higher phospho-tau accumulation (AT8) in S1 fractions from 9 to 12 month-old ThyTau22 mice compared to paired P301S mice. The relative abundance of phospho-tau was determined by densitometry analysis. Tau46 (low panel) was used as the loading control. Data is shown as the mean + SD (*n* = 3–6). ^∗^*p* < 0.05. Significance was analyzed by *t*-test between young and aged mice in each model and between aged P301S and ThyTau22 mice. **(B)** Representative western-blot of total tau accumulation (T46) in S1 and S3 fractions from WT, ThyTau22 and P301S mice. **(C)** Representative western-blot of phospho-tau (AT8 and AT100) in S1 and S1s fractions from P301S mice. **(D)** Quantitative analysis of BV2 viability (determined by flow cytometry) after 12 h of S1s treatment at 0.1 ug/ul. Data is shown as the mean + SD (*n* = 3).

In order to explore this possibility, we first quantified the phospho-tau forms in the soluble fractions from P301S and ThyTau22 mice. As shown in Figure 4A, a significant higher level of soluble (S1 fractions) phospho-tau forms (AT8 positive) was observed in ThyTau22 compared to P301S model. This finding, although unexpected, may explain the non-toxic effect on microglia cells of S1 fractions from 12 month-old P301S mice.

As reported above, there was a higher tau expression/accumulation in the P301S model than in the Thytau22 mouse model. Therefore, we hypothesized that most of tau forms in P301S mice could be aggregated and, in consequence, in form of insoluble tau species. Thus, we next evaluated the phospho-tau accumulation in different fractions isolated from mouse cortex (see Methods). Briefly, the S1 fractions comprise both extracellular and cytosolic proteins whereas the S3 fractions correspond to SDS releasable proteins. In consonance with our hypothesis, most (if not all) tau species were preferentially concentrated in the soluble fractions in ThyTau22 model whereas an appreciable fraction of tau was found in the SDS-releasable pool in P301S (see Figure 4B). Therefore, the lower toxicity and the lower soluble phospho-tau observed in P301S model could just reflect a higher aggregation status of the different phosphorylated Tau forms. In fact, aggregated phospho-tau (Sarkosyl-resistant fraction) extracted from either P301S model (showing a 96.8 ± 0.43% of BV2 viable cells after 12 h of treatment with Sarkosyl-resistant fraction, *n* = 3) or even from AD patients (Sanchez-Mejias et al., 2016) produced no microglial toxicity.

Finally, we evaluated whether the mechanical disaggregation of the phospho-tau fractions from P301S mice (see Methods) produced soluble and toxic phospho-tau species. To this effect, we sonicated and ultracentrifuge the pellet obtained after S1 fractions extraction (P1). The supernatants constituted the sonication-dependent soluble fractions (S1s). As expected, after sonication, soluble AT8- and AT100-positive bands were clearly visible in the S1s fractions (see Figure 4C). This AT8/AT100 positive band was absent in the non-sonicated S1 soluble fractions and was also absent at early ages, before tau phosphorylation. Therefore, sonication of the aggregated phospho-tau produced soluble phospho-tau forms.

Since our previous data indicated that soluble phospho-tau was toxic for microglia *in vitro* (Sanchez-Mejias et al., 2016), we then tested whether these S1s fractions were also toxic for BV2 cells. Unexpectedly, we did not observe any decrease in BV2 viability after 12 h of treatment with S1s fractions from 9 to 12 month-old P301S mice (see Figure 4D).

## Discussion

In this work we have compared the hippocampal microglial response in two different mutant transgenic tau mouse models, ThyTau22 and P301S. Surprisingly, our results demonstrate important differences between these two models of tau pathology. Contrary to the low microglial activation manifested in the hippocampus of ThyTau22 mice, the P301S mice display a strong microgliosis, even stronger than that observed in an amyloidogenic APP-model. In contrast, we also demonstrated that brain soluble S1 fractions isolated from ThyTau22 mice are highly toxic for microglial cells *in vitro*, whereas equivalent S1 fractions from P301S mice produce no toxicity. These discrepancies in the microglial response to tau pathology between both models may reflect differences in accumulation, phosphorylation and/or tau aggregation.

Numerous studies suggest that the exclusive presence of tau pathology induces microglial activation. In fact, microglial activation has been linked to tau deposition in different human tauopathies, such as AD (Sheffield et al., 2000), progressive supranuclear palsy (Ishizawa and Dickson, 2001) and corticobasal degeneration (Gerhard et al., 2004; Henkel et al., 2004), as well as in transgenic rats expressing human non-mutated truncated tau (Zilka et al., 2009; Stozicka et al., 2010), and in different transgenic mice with overexpression of the human mutated tau protein. In agreement with our results, a robust neuroinflammatory response characterized by microgliosis has been described in the mouse model expressing human P301S mutated tau protein under the control of the Thy1 promotor (Bellucci et al., 2004) and in patients with frontotemporal dementia carrying the P301S mutation (Bellucci et al., 2011). Additionally, the PS19 line, which is another transgenic mouse model carrying the P301S mutation, showed microgliosis and synaptic pathology in hippocampus preceding neurofibrillary tangle formation and neuronal loss (Yoshiyama et al., 2007). More recently, it has been stated that the age-dependent human mutated tau expression in the rTg4510 mouse model leads to tau-dependent inflammatory changes (Wes et al., 2014). In fact, the genetic removal of Tau seems to prevent the microglial activation and the associated neurodegeneration (Maphis et al., 2015a,b).

Moreover, microglial activation has been implicated in all the different pathological steps associated with tau-patholohy such as phosphorylation, aggregation, propagation and synaptic alteration (for review, see Laurent et al., 2018). It has recently been demonstrated that microglial cells phagocyte aggregated tau and secret exosomes, which are transmissible to neurons. In this sense, microglia are believed to play a key role in tau spreading (Asai et al., 2015).

However, as we and others have also reported (Sanchez-Mejias et al., 2016; Laurent et al., 2017) there is a mild microglial activation in some Tau-based models and even in AD samples. Present results also confirm the moderate microglial response in ThyTau22 model and the strong microglial activation in P301S mice. Similar differences between Tau models have been also reported using single cell RNA-seq (Friedman et al., 2018). The reasons that explain the distinct microglial responses are not known. The mechanisms underlying tau-mediated microglial activation has not yet been fully identified. Multiple membrane receptors and intracellular signal transduction pathways have been implicated, highlighting the essential role of TREM2 receptor (Takahashi et al., 2005; Melchior et al., 2010; Kleinberger et al., 2014). What is more, microglia from P301S mice showed higher TREM2 mRNA levels while the pathology is progressing, and silencing of TREM2 exacerbated tau pathology and spatial learning deficits in P301S mice (Jiang et al., 2015).

On the other hand, characterization of tau species involved in microglial activation is an open question. Morales et al. (2013) studied *in vitro* the effect of tau aggregates over microglial cells and concluded that oligomeric or filamentous tau forms, but not monomeric, triggered microglial activation. More recently, Nilson et al. (2017) demonstrated that tau oligomers take part in the feed-forward mechanisms, which leads to chronic neuroinflammation. They verified that T22-positive tau oligomers co-localized with microglia in frontotemporal lobar dementia and AD subjects, which may suggest the engulfment of tau oligomers by microglia and its consequent activation. According with these statements, and using the T22 antibody, we have confirmed, by dot-blot, the presence of oligomeric forms of tau in the soluble fraction S1 of the P301S hippocampus, but not in that of the ThyTau22 (data not shown), which could justify the remarkable differences in the microglial activation between these two tau models.

Additionally, the severity of tau pathology may be a key point in microglial activation. It has been reported that misfolded truncated tau protein is able to activate microglia at the concentration of 100 nM (Kovac et al., 2011). In support of this notion, our results demonstrated that the amount of total and phospho-tau species were much higher in aged P301S than in aged ThyTau22 mice. Moreover, the P301S hippocampus manifested a stronger and more extensive distribution of phospho-tau immunostaining than ThyTau22 mice. Similar results were found by Chen et al. (2016) in the bigenic line 5xFAD/ThyTau22. Therefore, the different microglial response could simply reflect the differences in tau expression between different models, as it is the case with APP-based models.

Beside the scarce microglial activation produced in the ThyTau22 transgenic model, we have previously reported a significant decrease in the hippocampal area covered by the individual microglia cells in 9–12 month-old animals. This microglial pathology could resemble that one present in the human Braak V–VI hippocampus, where most of the parenchymal space of the hilar region was not protected by microglial cells (Sanchez-Mejias et al., 2016). In the same sense, Streit et al. (2009) showed that neuronal structures positive for tau are associated with dystrophic (pathological) rather than activated microglial cells. We have also established that soluble phospho-tau was implicated in this degenerative microglial process in the AD hippocampus. Corroborating this fact, our *in vitro* results in this work demonstrated that the soluble (S1) fractions isolated from 12-month-old ThyTau22 mice, but not those from old P301S animals, were toxic for BV2 cells. More important, we demonstrated that the S1 fractions from aged ThyTau22 mice, compared to that from P301S mice, exhibited a much higher phospho-tau accumulation (AT8), which seems to be responsible for this microglial degeneration. In fact, our results confirmed that, at least extrinsic apoptotic pathway is mediating microglial death, as demonstrated by the significant activation of caspases 3 and 8 in BV2 cells after treatment with S1 from aged ThyTau22 mice.

Ours results established, for the first time, that in ThyTau22 model most tau species were preferentially concentrated in the soluble fractions whereas in P301S mice an appreciable fraction of tau seems to be aggregated in form of insoluble tau species. We have also detected that the insoluble fraction from 9 to 12 month-old P301S mice accumulated a higher content in phospho-tau species than the S1 fraction. However, and unexpectedly, these phospho-tau species, when solubilized, did not produced any toxic effect for microglia cells *in vitro*, in contrast to the degenerative effect of soluble phospho-tau from ThyTau22 mice and AD patients hippocampi. As these soluble fractions should contain a mixture of tau species, we cannot establish which form(s) of phospho-tau is directly involved in the toxic effect upon microglial cells. Nevertheless, it is important to highlight that not all phospho-tau species from AD samples are toxic for microglial cells, as demonstrated when treating BV2 cells with Sarkosyl-insoluble phospho-tau (Sanchez-Mejias et al., 2016). It is widely known that intracellular paired helical filaments, which are the basis for the formation of neurofibrillary tangles, content Sarkosyl-insoluble phospho-tau. Consequently, as it has already been stated, tangle formation may be considered as a protection mechanism which could prevent neuronal (Santacruz et al., 2005) and microglial toxicity.

In conclusion, our results show that tau pathology effect upon microglia differs enormously among transgenic tau models. A diversity of toxic tau species differing in morphology, solubility, and disease-relevant properties can be reported inresponse to different factors including tau mutations, isoform composition, and post-translational modification(Guo et al., 2017). These differing forms of phospho-tau might contribute to the distinct microglial responses observed in the two tauopathies murine models studied in this work. Consequently, not all mutant transgenic tau models reproduce the human AD-associated hippocampal microglial pathology. The molecular nature/conformation of the toxic tau species for microglial cells is an important issue to be addressed in future research.

## Author Contributions

CR-M, MV, JV, and AG drafted the manuscript. CR-M, MV, and JV designed the figures. CR-M, VN, RS-V, SJ, JF-V, MS-M, CM-C, AG, MV, and JV critically revised the manuscript for intellectual content. All authors approved the manuscript in its final form.

## Conflict of Interest Statement

The authors declare that the research was conducted in the absence of any commercial or financial relationships that could be construed as a potential conflict of interest.
